# Mental health and wellbeing coordinators in primary schools to support student mental health: protocol for a quasi-experimental cluster study

**DOI:** 10.1186/s12889-021-11467-4

**Published:** 2021-07-28

**Authors:** S. Darling, G. Dawson, J. Quach, R. Smith, A. Perkins, A. Connolly, A. Smith, C. L. Moore, J. Ride, F. Oberklaid

**Affiliations:** 1 Centre for Community Child Health, Murdoch Children’s Research Institute, Royal Children’s Hospital, 50 Flemington Rd, Parkville, VIC 3052 USA; 2 Department of Paediatrics, University of Melbourne, Melbourne, VIC 3010 USA; 3 Centre for Program Evaluation, Melbourne Graduate School of Education, University of Melbourne, Carlton, VIC 3053 USA; 4 Health Economics Unit, Centre for Health Policy, Melbourne School of Population and Global Health, 207 Bouverie St, Parkville, VIC 3010 USA

**Keywords:** Mental health, Paediatric, Primary school, Wellbeing, School capacity, Implementation

## Abstract

**Background:**

Half of mental health disorders begin before the age of 14, highlighting the importance of prevention and early-intervention in childhood. Schools have been identified globally by policymakers as a platform to support good child mental health; however, the majority of the research is focused on secondary schools, with primary schools receiving very little attention by comparison. The limited available evidence on mental health initiatives in primary schools is hindered by a lack of rigorous evaluation. This quasi-experimental cluster study aims to examine the implementation and effectiveness of a Mental Health and Wellbeing Co-ordinator role designed to build mental health capacity within primary schools.

**Methods:**

This is a primary (ages 5–12) school-based cluster quasi-experimental study in Victoria, Australia. Before baseline data collection, 16 schools selected by the state education department will be allocated to intervention, and another 16 matched schools will continue as ‘Business as Usual’. In intervention schools, a mental health and well-being coordinator will be recruited and trained, and three additional school staff will also be selected to receive components of the mental health training. Surveys will be completed by consenting staff (at 2-, 5-, 10- and 17-months post allocation) and by consenting parents/carers (at 3-, 10- and 17-months post allocation) in both intervention and business as usual schools. The primary objective is to assess the change in teacher’s confidence to support student mental health and wellbeing using the School Mental Health Self-Efficacy Teacher Survey. Secondary objectives are to assess the indirect impact on systemic factors (level of support, prioritisation of child mental health), parent and teachers’ mental health literacy (stigma, knowledge), care access (school engagement with community-based services), and student mental health outcomes. Implementation outcomes (feasibility, acceptability, and fidelity) and costs will also be evaluated.

**Discussion:**

The current study will examine the implementation and effectiveness of having a trained Mental Health and Wellbeing Coordinator within primary schools. If the intervention increases teachers’ confidence to support student mental health and wellbeing and builds the capacity of primary schools it will improve student mental health provision and inform large-scale mental health service reform.

**Trial registration:**

The trial was retrospectively registered in the Australian New Zealand Clinical Trials Registry (ANZCTR) on July 6, 2021. The registration number is ACTRN12621000873820.

## Background

It is estimated that 10–17% of children and young people aged 4–17 years meet criteria for a mental health condition [1–3], and half of mental health conditions begin by the age of 14 [4]. While for some children certain behaviours might be developmentally appropriate and resolve over time without intervention, in others they suggest mental health disorders that if left unaddressed can have deleterious impacts across every aspect of a child’s life. In the short-term, children with mental health conditions have higher rates of school absenteeism [5], resulting in poorer learning outcomes [6]. Longer-term, poor childhood mental health has been associated with increased rates of unemployment, violence, substance misuse, suicide, poorer social functioning and a lower quality of life in adulthood [7].

There has been a marked increase in presentations to tertiary services of children with non-urgent mental health issues over the past 13 years [8]. This is likely to reflect a combination of low child mental health literacy among parents (leading to delayed recognition of the existence and/or severity of the child’s mental health problem) and barriers to accessing appropriate, community-based early intervention services (i.e., service fragmentation, difficulty navigating the mental health system and high costs and long wait times to access specialist care) [2, 9–12]. In an effort to provide children with more appropriate and timely support before mental health problems become entrenched, there has been a strong push internationally to implement preventative approaches to mental health issues in children [13].

Schools have been consistently flagged by policymakers and other stakeholders as an ideal universal platform to promote and support good mental health for all students, to identify and manage students with emerging mental health issues early, and to target interventions for students with significant mental health problems [13–16]. For students with and without mental health issues, school-based mental health services have been identified as the most common setting for mental health support [17]. This is especially true for minority populations and for students with less obvious mental health issues (i.e., internalising or subclinical symptoms) who are less likely to seek out care [18–20].

In the adolescent age group, there have been different approaches both within Australia and other countries to increase the support provided in schools [13, 21]. Approaches which demonstrate impact in building the capacity of secondary schools include refining pathways to care [22], whole school approaches to changing school culture [23], mental health literacy programs for staff [24] and students [25–27], services provided by community-based counsellors and clinical practitioners [28, 29] and third party delivered intervention programs [30]. However, despite this significant progress in secondary schools, [8], child mental health and the role of primary (children aged between 5 and 12 years old) schools has received comparatively little attention. This is particularly problematic given the trend towards mental health issues emerging before the age of 14 [4]. Factors such as adults’ limited knowledge regarding child mental health (i.e., child mental health literacy), a reliance on parent and carer engagement and consent to access care and a fragmented child mental health system may all impede progress in child mental health prevention and support within the primary school setting [31].

This gap in primary schools has been recognised by educators themselves; they acknowledge that student mental health and wellbeing support is part of their core role but consistently report feeling overwhelmed by the volume and complexity of issues, a lack of mental health literacy and confidence in their ability to address students’ mental health issues, insufficient time, training and resources and poor access to specialist services [32–35]. In Australia, there have been national [36], state [37], and local examples [23] of programs designed to improve mental health support in primary schools. However, initiatives have either relied on untrained, time-poor school-based staff to take on additional tasks such as acting as a wellbeing officer, or clinicians from outside the school system to deliver services, often for only the most complex or severe cases [38, 39]. Neither of these approaches addresses the capacity and service access barriers cited by educators.

Many primary school mental health initiatives lack rigorous evaluation of a program’s effectiveness or how effectively it has been implemented in the school context [40]. Where there has been effort across the sector to evaluate programs, often this is done retrospectively or using cross sectional designs which fail to capture change over longer periods of time [41]. This has resulted in an incomplete evidence base regarding the sustainability of implementation and program outcomes. Further, the lack of a control comparison in many evaluations means it is not possible to conclude if the program has produced benefit over and above what is already occurring in the school. Only 33% of evidence-based programs are successfully implemented into practice [42], largely due to a lack of consideration paid to implementation strategies. To achieve successful, long-term sustainability of mental health initiatives in a complex multi-level system, it is important that key implementation principles be embedded within the design. In an education context, this requires program designers to consider not just the intervention and the individual, but also the *inner* (immediate school environment) and *outer settings* (region/district and the larger political, social and economic context) [43].

The primary school age period presents a unique opportunity to intervene early and modify the trajectory of many mental health issues and prevent progression to more chronic conditions. The intervention in this study has been designed to leverage this developmental window and address the gaps in the school-based child mental health system by introducing the concept of a Mental Health and Wellbeing Coordinator (MHWC) role in primary schools. The MHWC is an experienced qualified educator who will be an *additional* resource for the schools and will take up their role alongside participation in a comprehensive training program designed within an implementation science framework. The role and the training program combine to form the “The MHWC model”, which aims to build mental health capacity within the school. The MHWC will be trained to identify mental health issues, establish clear referral pathways, work proactively with other professionals, and implement whole-school approaches to mental health and wellbeing. This will equip schools and classroom teachers with an additional resource for supporting student mental health and wellbeing. In addition, the MHWC will act as the liaison between the school and community-based health and other community-based services.

The primary objective of this study is to assess whether the MHWC model leads to changes in classroom teachers’ self-reported confidence to support student mental health and wellbeing. Secondary objectives are to investigate the indirect impact on systemic factors (level of support, prioritisation of child mental health), parent and teachers’ mental health literacy (stigma, knowledge), care access (school engagement with services), and student mental health outcomes. Implementation outcomes (feasibility, acceptability, and fidelity) and costs will also be evaluated.

## Methods

### Design and setting

**Fig. 1:**
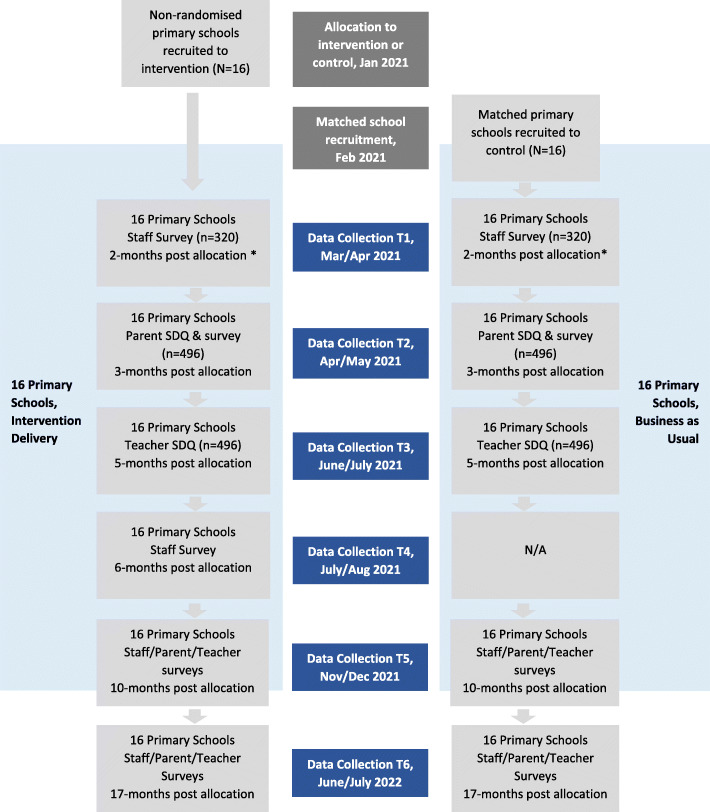
Flow chart estimating the progress of schools and participants through trial. *Staff surveys will be completed by MHWCs (intervention schools only) and school staff (school leaders, classroom teachers, education support staff)

The study is a quasi-experimental cluster study involving 16 intervention and 16 control (Business and Usual [BAU]) schools (Fig. 1). We will use a multi-method (qualitative and quantitative data), multi-site and multi-informant (MHWCs, school leaders, teachers, education support staff, parents/carers) design. This study has been approved by the Royal Children’s Hospital Human Research Ethics Committee (#65924) and the Victorian Department of Education and Training’s (DET) Research in Schools and Early Childhood settings (RISEC) process (#2020_004332).

iData collection will occur over six timepoints in both intervention and BAU schools beginning at timepoint 1 (T1; 2 months post allocation) and continuing through to timepoint 2 (T2, 3 months), timepoint 3 (T3, 5 months), timepoint 4 (T4; 6 months – intervention schools only), timepoint 5 (T5; 10 months) and timepoint 6 (T6; 17 months).

### Recruitment and participants

#### Schools

DET will recruit 16 intervention schools to receive the MHWC model from the North West (NW) and South West (SW) regions of Victoria, Australia in November 2020 (Fig. 1). Schools will be recruited by DET based on mental health need (through consultation with regional stakeholders and Incident Reporting Information System (IRIS) data), readiness (ensuring schools have the capacity and willingness to participate) and context diversity (including metropolitan, regional and rural contexts, and those impacted to different degrees by COVID-19). A comparison group of schools matched for socio-economic status (SES), location (metropolitan, regional, rural, remote) and number of student enrolments will be recruited by the research team and will act as controls (Business as Usual [BAU] schools). Secondary schools (ages 12–20), specialist schools (i.e., schools teaching specialist subjects or specialise teaching students with disability), and schools located outside NW, and SW regions of Victoria will be excluded. All participating schools will complete a formal consent process; details of what is required for schools to participate in the study will be outlined by the research team to the school Principal in a school plain language statement. An online consent form with the school plain language statement will be completed by the Principal or Assistant Principal prior to participant recruitment.

#### School staff

Across both intervention and BAU schools, classroom teachers, school leaders (principals, assistant principals, leading teachers) wellbeing staff and education support staff (i.e., non-teaching staff who provide student, teaching and/or school support) will be invited to participate in the study by completing an online survey at multiple timepoints (see Fig. 1 and Table 2). Staff within these roles will receive an email from their principal inviting them to complete an online survey. The email at T1 will include a link to complete a web-based consent form, including the relevant plain language statement, before completing the survey.

#### Years 2 and 4 classroom teachers and parents/carers

Across both intervention and BAU schools, parents/carers of students from years 2 and 4 (ages 7–8 and 9–10) will receive an email from the principal (T2) inviting them to (a) consent to information being collected about their child by the classroom teacher and (b) complete an online survey at two timepoints (see Fig. 1). Parents/carers must be the primary caregiver of a student from one of the selected year levels. Classroom teachers from years 2 and 4 will subsequently receive an email from the principal (T3) inviting them to complete the Strengths and Difficulties Questionnaire [52] on each child in their class for whom parental or carer consent has been obtained (see Fig. 1  and Table 2). Parents/carers and teachers will be asked to complete an online consent form, including the relevant plain language statement before completing the survey (Table 1).

**Table 1:** Summary of participant groups and projected number of participants in each group

Participant group		Number of participants	
		Intervention schools	BAU schools
MHWC and other trainees	MHWC	16 (1 per school)	–
	Training participants	48 (3 per school)	–
School staff	Classroom teachers^a^	192 (12 per school)	192 (12 per school)
	Education Support Staff	32–48 (at least 2 per school)	32–48 (at least 2 per school)
	School leadership (principal, APs, leading teachers, wellbeing)	32–48 (at least 2 per school)	32–48 (at least 2 per schools)
Parents/carers	Parents/carers from years 2 and 4	496 (31 per school)	496 (31 per school)
Year 2 / Year 4 classroom teachers	Classroom teachers from years 2 and 4	64 (2 classrooms per year level per school)	64 (2 classrooms per year level per school)

^a^ Primary outcome will be measured within this participant group. See further details under Sample Size Calculation

### Procedure

#### Intervention

The intervention will consist of the MHWC role and the MHWC training program.

#### Mental Health and wellbeing coordinator (MHWC) role

For each intervention school, a MHWC will be employed and funded a full-time equivalency (FTE; outlined below). To be recruited for this role the MHWC should have a teaching qualification and registration with the Victorian Institute of Teaching. Once recruited the MHWC will:

- Receive evidence-based training around supporting the mental health needs of primary school students;
- Embed evidence-based training and professional development (Tier 1 practices & frameworks) across the school and build the capability of teaching and education support staff to better identify and support students with mental health issues;
- Be a significant contributor to the school’s wellbeing team;
- Support the referral pathway for students identified as requiring further assessment and intervention within the school or to external community-based services (the MHWC role will not involve providing 1:1 counselling support to students);
- Work proactively with regional staff (i.e., psychologists, speech and language therapists, social workers), school wellbeing and leadership teams, and other health professionals (community-based psychologists, paediatricians, GPs, other allied health) to engage appropriate mental health support such as assessment, counselling, classroom based adjustments;
- Connect wellbeing initiatives across the school and be responsible for implementing whole school approaches to mental health and wellbeing, including the social and emotional learning curriculum.

MHWC full time equivalency (FTE) allocation will be determined based on number of student enrolments at each school using the following scale: 0–149 students = 0.4 FTE, 150–299 students = 0.6 FTE, 300–449 students = 0.8 FTE, > 449 students = 1.0 FTE. An additional loading will be provided for MHWCs working in regional and rural schools.

#### MHWC training program

The MHWC will participate in a purpose designed training program, delivered by the research team, to increase their knowledge, skills, and attitudes to effectively focus on building the capacity of the whole school, working with individual teachers and the whole staff cohort. To promote whole school upskilling, encourage staff buy-in, and maximise the chance of sustainably embedding the MHWC model in the broader school setting, leaders at each intervention school will nominate three additional staff members to attend components of the MHWC training (training participants). All MHWCs will receive an explanation of the research component at the induction session by the training delivery lead and complete a plain language statement. Nominated training participants must be members of school leadership (i.e., principal, assistant principal, leading teacher), wellbeing staff, or a teacher (classroom) at an intervention school. Administrative staff will be excluded from the MHWC or training participant groups.

**Fig. 2:**
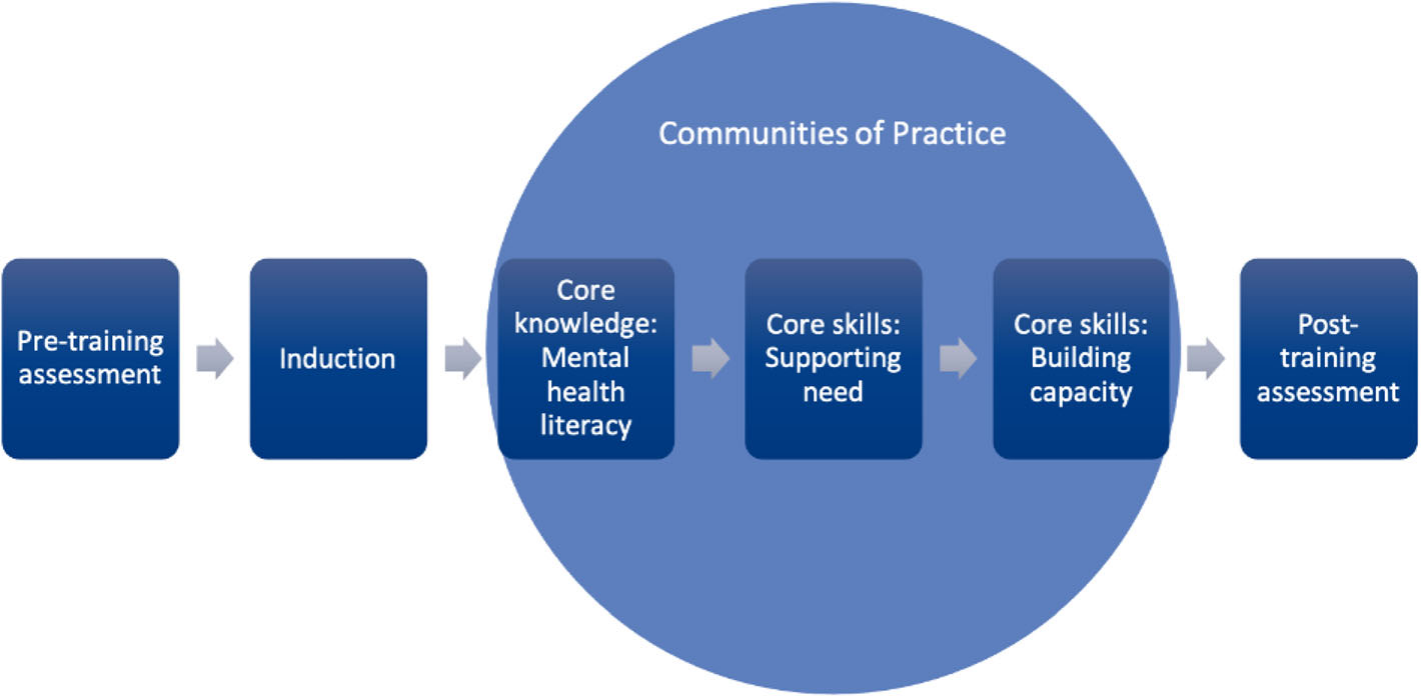
Content and structure of the training program for MHWCs

The intervention comprises an induction session followed by three core modules: Mental Health Literacy; Supporting Need; and Building Capacity attended by MHWCs and nominated additional school staff. MHWCs will also attend Communities of Practice sessions which will involve a selected expert for each session (Fig. 2). The content of the core modules will focus on the following broad areas:

- Mental health and wellbeing as a continuum, including behavioural, social emotional and learning indicators;
- Risk and protective factors for mental health issues and promoting wellbeing;
- Engaging parents and carers and supporting school staff in conversations with parents and carers about student mental health and wellbeing;
- Building and maintaining effective relationships with service providers;
- Understanding the referral pathways within the school system and into the community;
- Evidence-based prevention and promotion approaches and programs and evaluating their effectiveness and fit for purpose in the school context.

The instruction will use a problem/task centred approach informed by Merrill’s first principles of instruction [53]. These principles work on the basis that learning is promoted when: learners acquire knowledge in the context of real-world problems; existing knowledge is activated as a foundation for new knowledge; new knowledge is demonstrated to the learner, applied by the learner, and integrated into the learner’s world. MHWCs and other school participants will practice applying their knowledge through case studies, role-plays, and case studies and examples from their school context. Outputs of the training include a mental health and wellbeing profile and plan, and care pathway mapping based on each participant’s individual school context and circumstances.

Induction and the three core modules will be delivered over an intensive 3-day training program via a synchronous online environment, using teleconference technology to facilitate the building of professional networks and communities of practice between schools. In addition, asynchronous learning will include, videos, content-based activities, pre-reading, skills and knowledge checks, completion of online modules from other providers (as pre-requisites to cover basic concepts and engage with potential resources for staff professional development) and reviewing previously covered material to consolidate learning. Communities of practice and skills practice ‘on-the-job’ will also form an integral part of the learning approach and will continue as part of the model once the core training modules are delivered.

#### Comparison

BAU schools will not receive the intervention (MHWC role or training program). They will complete T1, T2, T3, T5 and T6 surveys (see Table 2).

**Table 2:** Summary of outcomes and measures

Outcome	Variable	Data source/Measure	Participant group						Data collection timepoint					
			MHWC	Trainee	School staff (INT)	School staff (BAU)	Years 2 / 4 Teachers	Years 2 / 4 Parents	T1	T2	T3	T4	T5	T6
Primary Outcome	Confidence to support student mental health and wellbeing	School Mental Health Self-efficacy Teacher Survey [44]	✔	✔	✔	✔			✔				✔	✔
Secondary Outcome	Perceived levels of support in managing child mental health	Study designed	✔	✔	✔	✔		✔	✔				✔	✔
	Mental health literacy	Child Health Poll [45] Study designed perceived confidence and skills Vignettes	✔	✔	✔	✔		✔	✔				✔	✔
	Child mental health stigma	The Attitudes about Child Mental Health Questionnaire [46]	✔	✔	✔	✔		✔	✔				✔	✔
	Student mental health and wellbeing	Strengths & Difficulties Questionnaire [47] Attitude to Schools Survey [48] and Parent Opinion Survey [49]					✔	✔		✔	✔		✔	✔
	Levels of engagement with DET-based and externally based mental health and wellbeing services	Study designed Attitude to Schools Survey [48] and Parent Opinion Survey [49]	✔	✔	✔	✔		✔	✔	✔			✔	✔
	Prioritisation of child mental health & wellbeing within school’s curriculum & planning	Study designed	✔	✔	✔	✔		✔	✔				✔	✔
	School engagement and perceived mental health support	Attitude to Schools Survey [48], Parent Opinion Survey [49] and study designed	✔	✔	✔	✔		✔	✔				✔	✔
	Level of unmet mental health and wellbeing need within classrooms	Study designed			✔	✔			✔				✔	✔
Process Outcomes	Implementation (feasibility, acceptability, appropriateness, reach, fidelity)	Acceptability of Intervention Measure, Intervention Appropriateness Measure & Feasibility of Intervention Measure [50] Focus Groups	✔	✔	✔								✔	
	Levels of engagement with MHWC	Study designed and Focus Groups	✔	✔	✔		✔					✔	✔	
	Cost	Cost inputs (time, resources, Casual Relief Teacher funding) Data will be obtained from referral activities log, job analysis, focus groups and surveys	✔						✔			✔	✔	
Moderators	Readiness to implement	Readiness to Implement scale [51]	✔	✔	✔				✔					
	MHWC Time allocation	FTE allocation; study devised measures	✔						✔			✔	✔	
	School & Participant Characteristics	Demographics	✔	✔	✔			✔	✔	✔				

## Primary and secondary outcomes and measures

### School and participant characteristics

#### School characteristics

Information will be collected for each school including the Index of Community Socio-Educational Advantage (ICSEA) [54] metropolitan, regional, rural or remote location as determined by DET, number of enrolments and region (NW or SW).

#### Participant demographic characteristics and professional experience

Basic demographic information will be collected via survey, including participant age, school postcode, highest level of education, languages spoken at home, the number of years teaching and/or working within primary schools (school staff only), previous participation in child mental health and wellbeing related training courses and professional development (school staff only).

### Primary outcome and measure

#### Teacher confidence in supporting mental health

The School Mental Health Self-Efficacy Teacher Survey (SMH-SETS) [44] will be completed by classroom teachers. The SMH-SETS is a psychometrically sound 15 item measure that utilises a 6-point Likert scale that addresses teacher’s confidence in their ability to successfully support students’ mental health with excellent internal consistency (Cronbach’s alpha = .91), item reliability and validity [55].

### Secondary outcomes and measures

#### Perceived level of support for student mental health

To evaluate the impact of the MHWC model on perceived levels of support for student mental health, study designed questions will be developed to capture (a) *how much support staff expect* in managing student mental health and wellbeing from within their school and DET, and (b) *how supported staff have felt in the past month* from within their school and DET.

#### Mental health literacy

To evaluate the perceived level of knowledge and confidence in identifying child mental health and wellbeing issues, study designed items will be developed and rated on a 6-point Likert scale from strongly agree to strongly disagree. Study devised multiple choice items requiring respondents to choose the correct answer will also be developed to capture knowledge about internalising and externalising mental health issues in students.

To evaluate skills and confidence related to mental health and wellbeing of students, school staff will complete a skills assessment at T1, T5 and T6 (see Table 2). The skills assessment will comprise case study vignettes covering a range of potential scenarios school staff may be presented with, and respondents will be asked about key behavioural issues, contributing factors, strategies and further support required, as well as confidence in supporting the child described in the vignette.

To evaluate parents/carers child mental health literacy, items will be drawn from the Child Health Poll – child mental health edition [45]. The Child Health Poll includes items that address parent confidence in recognising and managing child mental health issues (measured on a 3-point Likert scale: confident, somewhat confident, not confident) and items that address knowledge about child mental health (measured on a 6-point Likert scale: strongly agree to strongly disagree).

#### Child mental health stigma

To evaluate changes in attitudes and stigma the Attitudes about Child Mental Health Questionnaire (ACMHQ [46];) will be administered. It consists of 30 items with four subscales: child dangerousness/incompetence, general stereotypes, community devaluation/discrimination and personal attitudes. Each item is rated on a 6-point Likert response scale that ranges from 1 (strongly disagree) to 6 (strongly agree). The ACMHQ subscales have good to excellent internal consistency (α =0.78–0.96) [46].

#### Unmet mental health and wellbeing need

To evaluate changes in the level of unmet need for mental health and wellbeing support within school classrooms, study devised questions will be used to capture (a) the proportion of students within each teacher’s classroom who have unmet needs for school-based mental health and wellbeing support and (b) the barriers to obtaining mental health and wellbeing support for these students.

#### Child mental health and wellbeing

To evaluate the impact of the MHWC model on child mental health, the Strength and Difficulties Questionnaire (SDQ) will be used [47]. The 25-item measure is a brief emotional and behavioural screening questionnaire for children aged 4–10 years old. There are 5 subscales with 5 items in each; emotional symptoms, conduct problems, hyperactivity/inattention, peer relationships and prosocial behaviour. The SDQ has good concurrent and predictive validity, and satisfactory internal consistency [47].

#### Prioritisation of student mental health

To evaluate the level of prioritisation of child mental health and wellbeing, school leaders will answer questions exploring the level of priority given to wellbeing and mental health provision for students, whether wellbeing and mental health provision for students is part of the school strategic plan or annual implementation plan, or if the school has a policy related to child mental health and wellbeing.

#### Engagement with mental health support

School staff engagement with school-based and external child mental health services will be measured using study designed questions to capture the perceived level of engagement required, the actual level of engagement and the types of child mental health support and services provided to staff.

Student engagement with mental health support and perceived availability of mental health and wellbeing support will be measured using subscales from the Attitudes to School Survey (AToSS) years 4–6 version [48]. The AToSS is a DET administered survey that will be completed by students and consists of 60 items across 6 domains with 3–5 factors/subscales within each domain. The following subscales will be used: Teacher concern; School connectedness; Advocate at school; Managing bullying; Peer relationships; Perceptions of school; Social outcomes; Subjective physical & mental health; Experience of COVID-19. The survey items use a five-point Likert scale from 1 (strongly disagree) to 5 (strongly agree).

Parent engagement with school-based and external child mental health and wellbeing support will be measured using study designed items and items from the DET Parent Opinion Survey (POS) [49]. The following subscales from the POS will be used: Student agency and voice; Confidence & resiliency skills; Managing bullying; Promoting positive behaviour; School connectedness; Positive transitions; Remote & Flexible learning items (2020). The items use a five-point Likert scale from 1 (strongly disagree) to 5 (strongly agree).

### Implementation measures

#### Readiness to implement

To effectively evaluate the implementation of the MHWC model we will also seek to understand individual and contextual characteristics (across all participant groups) that are present prior to implementation which may also influence program outcomes. We will use the Readiness to Implement Scale [51] which looks at three key areas: feasibility, fit and staff support. It is a self-report measure, consisting of 20 items and has an overall reliability of Cronbach’s alpha [α] = .92 [51].

#### Costs of the intervention

Costs of delivering the intervention will be estimated to inform wider implementation. This will be based on budgets for each role, the log of activities kept by MHWCs tracking time and resources used and from records of replacement teachers filling in for classroom teachers to attend professional development and training activities run by the MHWCs. The costs will be from the perspective of the school.

#### MHWC referral activities log and job analysis

To evaluate the tasks, responsibilities, time, and resources required to achieve all aspects of the MHWC role and successful implementation of the MHWC model, MHWC activity will be recorded in a study developed database. This information will include number of students impacted, number of interactions with classroom teachers, time spent on tasks, type of mental health and wellbeing activities the MHWC is assisting with, number of referrals made, uptake/outcome of referral, waitlist times, number, and type of interactions with regional staff. This information will be collected for two working weeks during the academic school year (Semester 2).

#### Feasibility, appropriateness, and acceptability of the MHWC model

To evaluate the feasibility, appropriateness, and acceptability of the MHWC model we will use the Feasibility of Intervention Measure (FIM), Intervention Appropriateness Measure (IAM) and the Acceptability of Intervention Measure (AIM) [50] adapted as required. These measures will assess the extent to which a new treatment or innovation can be successfully used or carried out within a setting (FIM), the perceived fit, relevance and compatibility of the intervention for a given practice setting, provider, or consumer and/or perceived fit of the innovation to address a particular issue or problem (IAM) and the perception among implementation stakeholders that an intervention is agreeable or satisfactory (AIM). These measures consist of 4-items each targeting perceived intervention acceptability. Items are measured on a 5-point Likert scale (completely disagree-completely agree) and the score is a calculated mean. Each measure has excellent structural validity (FIM α = 0.89, IAM α = 0.91, AIM α = 0.85) and test-retest reliability (FIM α = 0.88, IAM α = 0.87, AIM α = 0.83) [50].

#### Engagement of school staff with MHWC model

To evaluate the level of engagement that school staff have with the MHWC model, questions will be developed to capture the perceived level of engagement required, the actual level of engagement and the types of support provided to staff by MHWCs.

#### Focus groups

To further explore and capture data regarding the feasibility, appropriateness, and acceptability of the model, purposive sampling will be used to recruit a subset of school and regional staff to participate in qualitative focus groups at T4 and T5, using a semi-structured interview guide. Focus groups will consist of between 3 and 8 participants and will include MHWCs and other trainees from participating intervention schools as well as a cross-section of other school staff (including leadership and education support staff, wellbeing staff, classroom teachers) and regional support staff. Interviews will be facilitated by an experienced qualitative researcher, audio-recorded with permission from the participants and transcribed verbatim using an external transcription service.

### Moderators

Exploration of potential moderators to the relationship between the intervention and the primary outcome will include planned FTE allocation of MHWC, socio-economic status (ICSEA value), school geographic location (metro/rural/regional), as well as process moderators; training dosage (hours attended of training) and local adjustments to MHWC FTE by the school during implementation (average hours worked in MHWC role).

### Sample size calculation

We based the sample size calculation on the primary study objective of detecting the impact of the MHWC model on classroom teachers’ self-reported confidence to support student mental health and wellbeing which is assessed by the School Mental Health Self-Efficacy Teacher Survey (SMH-SETS [55]). We assumed a mean SMH-SETS score of 67.7 (standard deviation = 9.5) in the BAU arm based on unpublished data provided by the measure authors [56]. Participation of 12 teachers per school with the intra-cluster (intra-school) correlation coefficient (ICC) set to 0.1 (expected ICC is unknown); the sample of 32 schools (16 intervention schools and 16 BAU schools) provides 80% power at the (2-sided) 5% level of significance to detect a mean difference between the trial arms of 3.9 on the teacher-reported SMH-SETs score.

### Planned statistical analysis

All analyses will be based on observed data only; i.e. we will assume data are Missing Completely At Random. We will make every attempt to ensure that data are not missing from surveys at the point of completion. If there are missing responses to surveys completed by school or regional staff, we will follow up via phone or email up to three times to obtain the missing responses. If missing data are > 10% for specific outcomes, then predictors of missing data will be explored and sensitivity analyses making further adjustments (i.e. valid under Missing At Random) will be used such as imputation. The details of how this will be conducted will be outlined in the statistical analysis plan.

The primary outcome (mean difference in SMH-SETS total score) will be analysed using mixed effects linear regression fitted at the teacher level, including a fixed effect for arm (MHWC vs. BAU) and a random effect for school, adjusting for baseline values of SMH-SETS and school matching criteria (ICSEA, number of enrolments and metro/regional/rural location).

According to the nature of the secondary outcomes to be analysed (binary, continuous or ordinal) the appropriate mixed effects model will be used to estimate the impact of the MHWC model on the outcome of interest compared to the BAU schools. These models will be fitted at the participant level, including a fixed effect for arm (MHWC vs. BAU) and a random effect for school adjusting for school matching criteria (ICSEA, number of enrolments and metro/regional/rural location) and baseline value of the outcome where available. Analyses will be done using Stata Statistical Software MP.

### Cost analysis

Variation in costs across participating schools will be evaluated to identify factors that may impact on the costs of delivering the intervention according to school characteristics such as number of student enrolments, location, and SES. Based on these estimated costs, budget impact of delivering the intervention state-wide will be calculated, with associated resource implications, particularly workforce.

### Qualitative analysis

Focus group transcripts will be deidentified and imported into the computer software package QSR Nvivo 12 [57]. Data analysis will follow the guidelines for reflexive thematic analysis [58]. An experienced qualitative researcher will independently code the data and emergent categories and themes will be cross-checked with the broader team for accuracy and to ensure the data is well represented. Study findings will be reported in line with the COREQ checklist for reporting qualitative research [59].

## Discussion

This study protocol introduces a quasi-experimental cluster study investigating the impact of a mental health and wellbeing coordinator (MHWC) model on classroom teacher confidence to support student mental health and wellbeing in an Australian primary school setting. Previous approaches have largely focussed on developing and evaluating specific mental health and wellbeing programs delivered using existing school resources in cross-sectional study designs with limited attention paid to implementation factors [38]. This protocol describes a novel approach to building the capacity of primary schools by introducing an additional resource for schools, the MHWC, which is embedded through an implementation science framework to maximise impact on target outcomes and long-term sustainability.

Despite the strengths of the proposed study, there are some practical limitations involved in conducting applied research in a school setting [60]. First, a randomised controlled trial (RCT), the gold standard for assessing effectiveness of an intervention, was not feasible in the current study as intervention schools were selected by DET based on mental health need, readiness and context diversity [61]. The quasi-experimental design does have the benefit of enabling enrolment of schools to the intervention based on motivation, which reflects the real-life situation of public health interventions. The lack of randomisation, however, can introduce both selection bias and confounding. We will aim to mitigate these biases by matching our control schools and adjusting for known confounders as well as T1 values at the analysis level.

Second, standardised measures and data collection methods for some of the project outcomes were not available (i.e., Mental Health Literacy [62], routine collection of outcomes (e.g. referral data)). Ideally standardised measures and data collection processes would be used to maximise the quality of the research conclusions, including comparisons with previous school-based mental health initiatives, and across intervention and BAU schools. This lack of measures relevant to the study outcomes and standardised processes for routine data collection in schools likely reflects a historical lack of scientific rigour in evaluating school-based initiatives [63]. To mitigate the lack of appropriate measures, the research team in collaboration with an expert advisory panel, developed bespoke items to target these outcomes. The measures developed for this project will likely be relevant for future projects and therefore measures development may form a sub-project of the broader study. To mitigate the lack of standardised, routine data collection across schools, the research team has developed data collection templates that will capture pertinent referral pathway information for students. Due to the absence of routinely collected resource or cost data on mental health supports in schools, it is not possible in this study to compare the costs in intervention and BAU schools, so the full cost implications of the model will not be estimated, nor will an economic evaluation that compares costs and outcomes between intervention and BAU schools be conducted.

Finally, there are additional context considerations in the state of Victoria in 2021 that will need to be taken into account during data analysis and interpretation. These include the acute and longer-term impacts of the COVID-19 pandemic, school closures and remote learning on the mental health of students, teachers and the education community [64, 65] as well as the resources and programs being implemented by individual schools following the findings and recommendations of the Royal Commission into Victoria’s Mental Health System in March 2021 [66]. The COVID-19 pandemic is a fluid situation, so that a comprehensive risk mitigation strategy has been developed to plan for various situations as they arise (e.g., higher than anticipated attrition rate for schools and participants, schools returning to remote learning and the MHWC role and training being implemented using teleconferencing methods). In contrast, the findings from the Royal Commission are static, but may have longer-term, permanent implications for the offerings provided by schools to better support student mental health. To mitigate this, a comprehensive list of actions taken in response to the Royal Commission recommendations will be collected for each school and taken into account when interpreting data from the proposed study.

Results from this study will inform decision-making around large-scale mental health service reform and possible state and national roll out of the intervention [66]. To ensure the ongoing quality and sustainability of the MHWC model should it prove to be efficacious, it is imperative that there is ongoing monitoring and evaluation of the model is conducted. This would involve follow up of primary and secondary outcomes as well as implementation factors associated with the MHWC model including identification of enablers and barriers across school, region/district and system levels [43]. This design will address some of the shortcomings of previous approaches to address child mental health issues in primary schools and maximise the likelihood that the MHWC intervention will have positive long-term, sustainable effects on child mental health. This approach will not only impact the individual child and their family, but benefits may also be seen at a community and societal scale, across education and health sectors, and likely be experienced by generations to come.

## Data Availability

Not applicable.

## Abbreviations

ACMHQ: Attitudes about Child Mental Health Questionnaire
AIM: Acceptability of Intervention Measure
AToSS: Attitudes to School Survey
BAU: Business of Usual
FTE: Full-Time Equivalent
ICSEA: Index of Community Socio-Educational Advantage
MHWC: Mental Health and Wellbeing Coordinator
NW: North-West
POS: Parent Opinion Survey
RCT: Randomised Control Trial
SES: Socio-Economic Status
SQD: Strengths and Difficulties Questionnaire
SMH-SETS: School Mental Health Self-Efficacy Teacher Survey
SW: South-West

## References

[1] Green H, Mcginnity A, Meltzer, Ford T, Goodman R. Mental Health of Children and Young People in Great Britain: 2004. London: Office for National Statistics; 2005.

[2] Lawrence D, Johnson S, Hafekost J, Boterhoven de Haan K, Sawyer M, Ainley J, Zubrick SR (2015). The mental health of children and adolescents: Report on the second Australian child and adolescent survey of mental health and wellbeing.

[3] Whitney DG, Peterson MD (2019). US national and state-level prevalence of mental health disorders and disparities of mental health care use in children. JAMA Pediatr.

[4] Kessler RC, Amminger GP, Aguilar-Gaxiola S, Alonso J, Lee S, Ustun TB (2007). Age of onset of mental disorders: a review of recent literature. Curr Opin Psychiatry.

[5] Lawrence D, Dawson V, Houghton S, Goodsell B, Sawyer MG (2019). Impact of mental disorders on attendance at school. Australian J Educ.

[6] Hancock KJ, Shepherd CC, Lawrence D, Zubrick SR (2013). Student attendance and educational outcomes: Every day counts.

[7] Patel V, Flisher AJ, Hetrick S, McGorry P (2007). Mental health of young people: a global public-health challenge. Lancet.

[8] Hiscock H, Neely RJ, Lei S, Freed G (2018). Paediatric mental and physical health presentations to emergency departments, Victoria, 2008–15. Med J Aust.

[9] Paton K, Gillam L, Warren H, Mulraney M, Coghill D, Efron D, et al. Clinicians’ perceptions of the Australian Paediatric mental Health service system: problems and solutions. Aust N Z J Psychiatry. 2021; 4867420984242. 10.1177/0004867420984242 33461341

[10] Reardon T, Harvey K, Baranowska M, O’Brien D, Smith L, Creswell C (2017). What do parents perceive are the barriers and facilitators to accessing psychological treatment for mental health problems in children and adolescents? A systematic review of qualitative and quantitative studies. Eur Child Adolesc Psychiatry.

[11] Sawyer MG, Kosky RJ, Graetz BW, Arney F, Zubrick SR, Baghurst P (2000). The National Survey of mental Health and wellbeing: the child and adolescent component. Aust N Z J Psychiatry.

[12] Mulraney M, Lee C, Freed G, Sawyer M, Coghill D, Sciberras E, Efron D, Hiscock H (2021). How long and how much? Wait times and costs for initial private child mental health appointments. J Paediatr Child Health.

[13] UK Department of Health and Education (2017). Transforming Children and Young People’s Mental Health Provision: a Green Paper.

[14] Lourie IS, Hernandez M (2003). A historical perspective on national child mental health policy. J Emot Behav Disord.

[15] Commission (2020). P. Mental Health, Report no. 95.

[16] Psychologists NAoS. Communication planning and message development: Promoting school-based mental health services. Communique. 2006;35(1).

[17] Duong MT, Bruns EJ, Lee K, Cox S, Coifman J, Mayworm A, et al. Rates of mental Health service utilization by children and adolescents in schools and other common service settings: a systematic review and meta-analysis. Admin Pol Ment Health. 2020;48(3):420-439. 10.1007/s10488-020-01080-9 32940884

[18] Foster EM, Conner T (2005). Public costs of better mental health services for children and adolescents. Psychiatr Serv.

[19] Kataoka SH, Stein BD, Jaycox LH, Wong M, Escudero P, Tu W, Zaragoza C, Fink A (2003). A school-based mental health program for traumatized Latino immigrant children. J Am Acad Child Adolesc Psychiatry.

[20] Stephan SH, Weist M, Kataoka S, Adelsheim S, Mills C (2007). Transformation of children’s mental health services: the role of school mental health. Psychiatr Serv.

[21] Victorian Department of Education and Training (2020). Mental health practitioners in secondary schools.

[22] Wei Y, Kutcher S, Szumilas M (2011). Comprehensive school mental Health: an integrated “school-based pathway to care” model for Canadian secondary schools. McGill J Educ.

[23] Patton GC, Glover S, Bond L, Butler H, Godfrey C, Pietro GD, Bowes G (2000). The gatehouse project: a systematic approach to mental health promotion in secondary schools. Aust N Z J Psychiatry..

[24] Yamaguchi S, Foo JC, Nishida A, Ogawa S, Togo F, Sasaki T (2020). Mental health literacy programs for school teachers: a systematic review and narrative synthesis. Early Interv Psychiatry.

[25] Naylor PB, Cowie HA, Walters SJ, Talamelli L, Dawkins J (2009). Impact of a mental health teaching programme on adolescents. Br J Psychiatry.

[26] Hart LM, Cropper P, Morgan AJ, Kelly CM, Jorm AF (2020). Teen mental Health first aid as a school-based intervention for improving peer support of adolescents at risk of suicide: outcomes from a cluster randomised crossover trial. Aust N Z J Psychiatry..

[27] Perry Y, Petrie K, Buckley H, Cavanagh L, Clarke D, Winslade M, Hadzi-Pavlovic D, Manicavasagar V, Christensen H (2014). Effects of a classroom-based educational resource on adolescent mental health literacy: a cluster randomised controlled trial. J Adolesc.

[28] Rickwood D, Paraskakis M, Quin D, Hobbs N, Ryall V, Trethowan J, McGorry P (2019). Australia’s innovation in youth mental health care: the headspace Centre model. Early Interv Psychiatry..

[29] Hetrick SE, Bailey AP, Smith KE, Malla A, Mathias S, Singh SP, O’Reilly A, Verma SK, Benoit L, Fleming TM, Moro MR (2017). Integrated (one-stop shop) youth health care: best available evidence and future directions. Med J Aust.

[30] Michelson D, Malik K, Parikh R, Weiss HA, Doyle AM, Bhat B, Sahu R, Chilhate B, Mathur S, Krishna M, Sharma R, Sudhir P, King M, Cuijpers P, Chorpita B, Fairburn CG, Patel V (2020). Effectiveness of a brief lay counsellor-delivered, problem-solving intervention for adolescent mental health problems in urban, low-income schools in India: a randomised controlled trial. Lancet Child Adolesc Health.

[31] Darling S, Oberklaid F. Child mental health: Building a shared language. MJA Insight+. 2019; Available from: https://insightplus.mja.com.au/2019/36/child-mental-health-building-a-shared-language/.

[32] Roeser R, Midgley C (1997). Teachers’ views of issues involving students’ mental health. Elem Sch J.

[33] Reinke WM, Stormont M, Herman KC, Puri R, Goel N (2011). Supporting children’s mental health in schools: teacher perceptions of needs, roles, and barriers. Sch Psychol Q.

[34] Graham A, Phelps R, Maddison C, Fitzgerald R (2011). Supporting children’s mental health in schools: teacher views. Teachers Teaching.

[35] Patalay P, Giese L, Stankovic M, Curtin C, Moltrecht B, Gondek D (2016). Mental health provision in schools: priority, facilitators and barriers in 10 European countries. Child Adolesc Ment Health..

[36] Beyond Blue (2020). Beyond Blue welcomes the Commonwealth’s renewed support of Be You.

[37] Victorian Department of Education and Training (2021). Respectful Relationships.

[38] Gee B, Wilson J, Clarke T, Farthing S, Carroll B, Jackson C, King K, Murdoch J, Fonagy P, Notley C (2021). Review: delivering mental health support within schools and colleges - a thematic synthesis of barriers and facilitators to implementation of indicated psychological interventions for adolescents. Child Adolesc Ment Health.

[39] Slade EP (2002). Effects of school-based mental health programs on mental health service use by adolescents at school and in the community. Ment Health Serv Res.

[40] Dawson G, Quach J, Smith A, Giles-Kay A, Loschiavo K, Oberklaid F, Goldfeld S, Sanci L, Reavley N, Williams I (2020). Interim report: development of the evidence evaluation criteria for the be you programs directory.

[41] Littlefield L, Cavanagh S, Knapp R, O’Grady L (2017). KidsMatter: Building the capacity of Australian primary schools and early childhood services to foster children’s social and emotional skills and promote children’s mental health.

[42] Damschroder LJ, Aron DC, Keith RE, Kirsh SR, Alexander JA, Lowery JC (2009). Fostering implementation of health services research findings into practice: A consolidated framework for advancing implementation science. Implement Sci..

[43] Lyon AR, Bruns EJ (2019). From evidence to impact: joining our best school mental health practices with our best implementation strategies. School Ment Health.

[44] Brann KL, Boone WJ, Splett JW, Clemons C, Bidwell SL. Development of the school mental Health self-efficacy teacher survey using Rasch analysis. J Psychoeduc Assess. 2020; 0734282920947504.

[45] Health CCC (2012). Children’s mental health. Policy Brief 24.

[46] Heflinger CA, Wallston KA, Mukolo A, Brannan AM (2014). Perceived stigma toward children with emotional and behavioral problems and their families: the attitudes about child mental Health questionnaire (ACMHQ). J Rural Ment Health.

[47] Goodman R (2001). Psychometric properties of the strengths and difficulties questionnaire. J Am Acad Child Adolesc Psychiatry.

[48] Victorian Department of Education and Training (2021). Data Collection and Surveys - Attitudes to School Survey.

[49] Victorian Department of Education and Training (2021). Data Collection and Surveys - Parent Opinion Survey.

[50] Weiner BJ, Lewis CC, Stanick C, Powell BJ, Dorsey CN, Clary AS, Boynton MH, Halko H (2017). Psychometric assessment of three newly developed implementation outcome measures. Implement Sci.

[51] Bliss CM, Wanless SB (2018). Development and initial investigation of a self-report measure of teachers’ readiness to implement. J Educ Chang.

[52] Goodman R (1997). The strengths and difficulties questionnaire: a research note. J Child Psychol Psychiatry.

[53] Merrill MD (2002). First principles of instruction. Educ Technol Res Dev.

[54] Authority ACAaR (2014). ICSEA 2013: technical report.

[55] Brann KL, Boone WJ, Splett JW, Clemons C, Bidwell SL (2021). Development of the school mental Health self-efficacy teacher survey using Rasch analysis. J Psychoeduc Assess.

[56] Brann K (2020). School mental Health self-efficacy teacher survey: unpublished data.

[57] QSR International Pty Ltd (2021). NVivo qualitative data analysis Software. Version 12.0.

[58] Braun V, Clarke V (2013). Successful qualitative research: a practical guide for beginners: sage.

[59] Tong A, Sainsbury P, Craig J (2007). Consolidated criteria for reporting qualitative research (COREQ): a 32-item checklist for interviews and focus groups. Int J Qual Health Care.

[60] Conroy MA, Stichter JP, Daunic A, Haydon T (2008). Classroom-based research in the field of emotional and behavioral disorders. J Spec Educ.

[61] Camm AJ, Fox KAA (2018). Strengths and weaknesses of ‘real-world’ studies involving non-vitamin K antagonist oral anticoagulants. Open Heart.

[62] O'Connor M, Casey L, Clough B (2014). Measuring mental health literacy--a review of scale-based measures. J Ment Health.

[63] Kutcher S, Wei Y, Behzadi P (2017). School-and community-based youth suicide prevention interventions: hot idea, hot air, or sham?. Can J Psychiatry.

[64] Loades ME, Chatburn, E., Higson-Sweeney, N., Reynolds, S., Shafran, R., Brigden, A., Linney, C., McManus, M.N., Borwick, C. and Crawley, E. Rapid systematic review: the impact of social isolation and loneliness on the mental health of children and adolescents in the context of COVID-19. J Am Acad Child Adolesc Psychiatry 2020;59(11):1218–1239, DOI: 10.1016/j.jaac.2020.05.009. 10.1016/j.jaac.2020.05.009 PMC7267797 32504808

[65] Baker CN, Peele H, Daniels M, Saybe M, Whalen K, Overstreet S, et al. The experience of COVID-19 and its impact on teachers’ mental Health, coping, and teaching. Sch Psychol Rev. 2021:1–14. 10.1080/2372966X.2020.1855473.

[66] State of Victoria. Collaboration to support good mental health and wellbeing. Royal Commission into Victoria’s Mental Health System, Final Report. Victoria: Royal Commission into Victoria's Mental Health System; 2021.

